# Efficiency of a mechanical device in controlling tracheal cuff pressure in intubated critically ill patients: a randomized controlled study

**DOI:** 10.1186/s13613-015-0054-z

**Published:** 2015-06-02

**Authors:** Saad Nseir, Andrey Rodriguez, Paula Saludes, Julien De Jonckheere, Jordi Valles, Antonio Artigas, Ignacio Martin-Loeches

**Affiliations:** Critical Care Center, Hospital de Sabadell, Universidad Autonoma de Barcelona, CIBER enfermedades respiratorias, Corporació Sanitaria Parc Tauli, Parc Tauli 1, 08208 Sabadell, Spain; Critical Care Center, R. Salengro Hospital, CHRU de Lille, rue E. Laine, 59037 Lille Cedex, France; Clinical Investigation Center—Innovative Technologies, INSERM CIC-IT 807, University Hospital of Lille, 152 rue du Dr. Yersin, 59120 Loos, France; Multidisciplinary Intensive Care, St James’s University Hospital, Dublin, Ireland

**Keywords:** Cuff pressure, Control, Tracheal ischemia, Microaspiration, Mechanical ventilation

## Abstract

**Background:**

Cuff pressure (*P*_cuff_) control is mandatory to avoid leakage of oral secretions passing the tracheal tube and tracheal ischemia. The aim of the present trial was to determine the efficacy of a mechanical device (PressureEasy®) in the continuous control of *P*_cuff_ in patients intubated with polyvinyl chloride (PVC)-cuffed tracheal tubes, compared with routine care using a manometer.

**Methods:**

This is a prospective, randomized, controlled, cross-over study. All patients requiring intubation with a predicted duration of mechanical ventilation ≥48 h were eligible. Eighteen patients randomly received continuous control of *P*_cuff_ with PressureEasy® device for 24 h, followed by discontinuous control (every 4 h) with a manual manometer for 24 h, or vice versa. *P*_cuff_ and airway pressure were continuously recorded. *P*_cuff_ target was 25 cmH_2_O during the two periods.

**Results:**

The percentage of time spent with *P*_cuff_ 20–30 cmH_2_O (median (IQR) 34 % (17–57) versus 50 % (35–64), *p* = 0.184) and the percentage of time spent with *P*_cuff_ <20 cmH_2_O (23 % (5–63) versus 43 % (16–60), *p* = 0.5) were similar during continuous control of *P*_cuff_ and routine care, respectively. However, the percentage of time spent with *P*_cuff_ >30 cmH_2_O was significantly higher during continuous control compared with routine care of tracheal cuff (26 % (14–39) versus 7 % (1–18), *p* = 0.002). No significant difference was found in *P*_cuff_ (25 (18–28) versus 21 (18–26), *p* = 0.17), mean airway pressure (14 (10–17) versus 14 (11–16), *p* = 0.679), or coefficient of variation of *P*_cuff_ (19 % (11–26) versus 20 % (11–25), *p* = 0.679) during continuous control compared with routine care of tracheal cuff, respectively.

**Conclusions:**

PressureEasy® did not demonstrate a better control of *P*_cuff_ between 20 and 30 cmH_2_O, compared with routine care using a manometer. Moreover, the device use resulted in significantly higher time spent with overinflation of tracheal cuff, which might increase the risk for tracheal ischemic lesions.

**Trial registration:**

Clinicaltrial.gov: NCT02109003

## Background

Intubation is an invasive procedure that is still performed in a large proportion of critically ill patients [1]. Some long-term intubation-related complications are caused by inappropriate cuff pressure (*P*_cuff_), and include microaspiration, and tracheal ischemic lesions [2, 3]. Microaspiration of contaminated oropharyngeal secretions is the main route of entry for bacteria into the lower respiratory tract [4, 5]. Colonization of the lower respiratory tract could progress into ventilator-associated pneumonia (VAP) when local or general defense mechanisms are altered in intubated critically ill patients [6]. VAP is the most frequent infection acquired in the ICU and is associated with high morbidity and mortality, especially in patients with comorbidities [7]. Tracheal ischemic injury is also associated with high morbidity, especially when routine care for tracheal cuff is not adequately provided [8–11].

Based on international recommendations, *P*_cuff_ should be kept between 20 and 30 cmH_2_O using a manometer [12, 13]. However, several recent studies suggested that a noncontinuous control of *P*_cuff_ using a manometer is not effective in intubated critically ill patients [14]. Further, previous studies did not identify modifiable risk factors for overinflation or underinflation of tracheal cuff [15–18].

Recently, several devices allowing continuous control of *P*_cuff_ were evaluated [19–22]. These devices are classified into pneumatic (i.e., does not require power supply, but a single-use 200-ml cylindrical cuff) and electronic devices (requiring power supply). Two prospective randomized controlled animal and human studies first validated the use of a pneumatic device [19, 20]. A noncommercially available device has also been validated and proved to be efficient in continuously controlling *P*_cuff_ in ICU patients [22]. However, other commercially available automated devices were only validated in in vitro studies [23, 24]. Recent data suggest that these devices interfere with the self-sealing characteristics of high-volume low-pressure (HVLP) tracheal cuffs [23]. Further, these electronic devices have been shown to be less efficient than the pneumatic device in continuous control of *P*_cuff_, because of rapid correction of overinflation episodes [21].

A new mechanical device with an improved design aiming at continuously controlling *P*_cuff_ has been commercialized (PressureEasy®, Smiths medical). The advantages of using such device are its small size and lower cost compared with other devices. However, to our knowledge, no study has evaluated the efficiency of this device in controlling *P*_cuff_. Therefore, we conducted this prospective randomized cross-over study to determine the efficiency of this device in continuously controlling *P*_cuff_. Our hypothesis was that this mechanical device would allow significant reduction of time spent with underinflation or overinflation of *P*_cuff_, compared with routine care, using a manual manometer.

## Methods

### Ethical aspects

This prospective randomized controlled study was performed in a 16-bed ICU of the teaching hospital of Sabadell (Spain), from April 2014 to July 2014. The institutional review board of the Parc Tauli University Hospital approved the study. Written consent was obtained from the patients or from their next of kin (ClinicalTrials.gov identifier: NCT02109003 http://clinicaltrials.gov/show/NCT02109003).

### Inclusion and exclusion criteria

All patients older than 18 years who were intubated with a predicted duration of mechanical ventilation ≥48 h were eligible for the study. Patients were excluded if they (1) were admitted to the ICU with a previous tracheostomy, (2) were enrolled in another study that might influence this study results, or (3) were intubated and mechanically ventilated >48 h at the time of randomization.

### Randomization

**Fig. 1:**
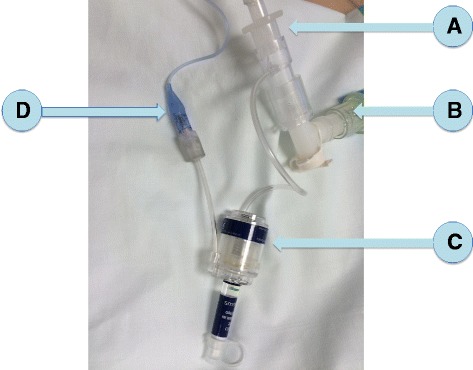
The PressureEasy® device. **a** Tracheal tube, (**b**) ventilator circuit, (**c**) PressureEasy®, and (**d**) external tracheal cuff

Patients were randomly assigned to receive continuous control of *P*_cuff_ with the mechanical device (PressureEasy®) (Fig. 1) for 24 h, followed by discontinuous control (every 4 h) with a manual manometer (Hi-Lo Hand Pressure Gauge, Covidien, TM, Malinckrodt TM) for 24 h; or discontinuous control of *P*_cuff_ followed by continuous control of *P*_cuff_. The target of *P*_cuff_ was 25 cmH_2_O during the two periods. Randomization was performed using a computer-generated random assignment list in balanced blocs of four. Treatment assignments were contained in sealed opaque envelopes sequentially numbered.

PressureEasy® is a single-patient use device, designed to continuously monitor tracheal *P*_cuff_. Its indicator window signals that *P*_cuff_ is maintained between 20 and 30 cmH_2_O. In addition, the airway pressure auto-feedback feature boosts *P*_cuff_ to ensure proper sealing when high pressures are used during ventilation. A pressure feedback line is designed to eliminate cuff leaks at peak inspiratory pressure.

### Outcome measurement

**Fig. 2:**
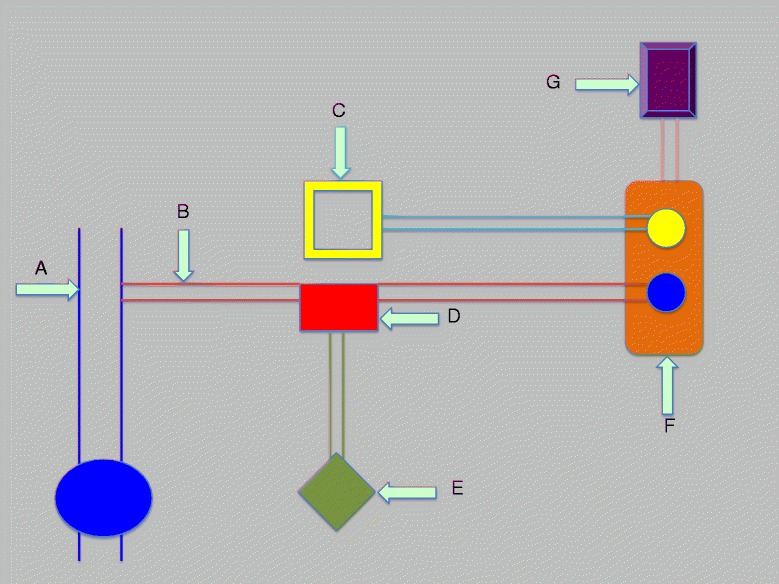
Description of connections between pressure transducers, ventilator, and tracheal tube. **a** Tracheal tube, (**b**) connection between external cuff and the three-way stopcock, (**c**) ventilator, (**d**) three-way stopcock, (**e**) PressureEasy® device or manometer, (**f**) pressure transducers connected to the ventilator and to tracheal cuff, and (**g**) laptop

In order to determine the percentage of patients with underinflation and overinflation of tracheal cuff and the duration of these episodes, the *P*_cuff_ and the airway pressure were continuously recorded at a digitizing frequency of 100 Hz for 48 h (Physiotrace®; Estaris, Lille, France) [25], including 24 h of continuous control of *P*_cuff_ using the mechanical device and 24 h of manual control of *P*_cuff_ using the manometer. The connection between the pressure transducer and the tracheal cuff was identical in the two study periods, with a three-way stopcock of which the third port was either connected to the mechanical device or closed and connected to the manometer every 4 h (Fig. 2). A second pressure transducer was connected to the heat and moisture exchanger, in order to record respiratory pressure. Pressure transducers were connected to a laptop, in order to continuously transfer pressure-time curves and mean values. Connections were checked every 4 h. The engineer who analyzed the data (JDJ), using the same program, was blinded to the randomization order.

### Study population

All the patients were intubated with a high-volume low-pressure PVC-cuffed tracheal tube with continuous subglottic secretion drainage (TaperGuard Evac, Covidien, Mallinckrodt) and were included within the first 48 h of intubation. Tracheal tube size was 8 and 7.5 in men and women, respectively. During the manometer period, nurses adjusted *P*_cuff_ every 4 h. Tracheal suctioning was performed six times a day or more frequently if clinically indicated.

### Other definitions

Underinflation of tracheal cuff was defined as *P*_cuff_ <20 cmH_2_0 for >5 min over the 24-h period of recording. Overinflation of tracheal cuff was defined as *P*_cuff_ >30 cmH_2_0 for >5 min over the 24-h period of recording [14].

The primary objective was to determine the efficiency of the mechanical device in reducing the percentage of time spent with underinflation or overinflation of tracheal cuff, compared with routine care using a manometer. The secondary objective was to determine the efficiency of the mechanical device in reducing the percentage of patients with underinflation or overinflation of tracheal cuff, compared with routine care.

The coefficient of variation of *P*_cuff_ was calculated as standard deviation/mean *P*_cuff_ × 100.

### Statistical analyses

#### Sample size calculation

Based on unpublished preliminary results, the mean percentage of time spent with underinflation or overinflation of tracheal cuff was 30 % (SD 20 %) in patients intubated with a PVC-cuffed tracheal tube receiving routine care of *P*_cuff_ using a manual manometer. The expected mean percentage of time with underinflation or overinflation of tracheal cuff using the mechanical device was 10 % (expected difference of 20 %). Considering a power of 90 % and an alpha risk of 5 %, the inclusion of 22 patients was required in a parallel-group design. However, when we took into account the cross-over design, the number of patients to include was 18.

#### Result analysis

All analyses were performed in an intention-to-treat manner. Distribution of quantitative variables was tested using the Shapiro-Wilk test. Normally and nonnormally distributed variables were expressed as mean ± SD and median (25th, 75th interquartile), respectively. The difference between the two groups was considered as significant if *p* < 0.05. McNemar and Wilcoxon tests were used to compare qualitative and quantitative variables between the two 24-h periods, respectively.

We calculated the median (25th, 75th interquartile) value of mean airway pressure in all patients and considered patients with airway pressure >75th interquartile as patients with high airway pressure. In order to determine the impact of high airway pressure on the efficiency of the mechanical device, we repeated all statistical analyses after exclusion of patients with high airway pressure.

## Results

### Patient characteristics

Eighteen patients were included in this study. Their mean age was 62 ± 12 years. At ICU admission, SAPS II and SOFA scores were 51 ± 14 and 7.7 ± 3.1, respectively. Duration of mechanical ventilation and ICU stay were 25 ± 18 days and 33 ± 21 days, respectively. No significant difference was found between the two study periods regarding the percentage of patients receiving sedation or neuromuscular-blocking agents. Glasgow Coma score, SOFA score, and PEEP were also similar during the two periods (Table 1). All patients received assist-control ventilation during the recording period. No significant difference was found in mean airway pressure between the two study periods (Table 2.)

**Table 1:** Patient characteristics during the 48 h following randomization

Variables	Continuous control of *P*_cuff_	Routine care	*p* values
	*n* = 18	*n* = 18	
SOFA score	8.8 ± 3.4	9 ± 3.7	0.458
Sedation	14 (77)	14 (77)	>0.999
Neuromuscular-blocking agent use	1 (5)	0 (0)	>0.999
Glasgow coma score^a^	7.5 ± 3	9 ± 3.7	0.137
PEEP	6.7 ± 1.9	6.6 ± 1.9	0.157

Data are frequencies (%) or mean ± SD

*SOFA* sequential organ failure assessment, *PEEP* positive end-expiratory pressure

^a^The verbal response was evaluated as in intubated patients: 1 no answer, 2 seems able to give simple responses, and 5 seems able to speak

**Table 2:** Impact of the mechanical device on tracheal cuff pressure

Variables	Continuous control of *P*_cuff_	Routine care	*p* values
	*n* = 18	*n* = 18	
Mean airway pressure, cmH_2_O	14 (10–17)	14 (11–16)	0.679
Mean *P*_cuff_, cmH_2_O	25 (18, 28)	21 (18, 26)	0.17
Coefficient of variation of *P*_cuff_, %	19 (11, 26)	20 (12, 25)	0.67
*P*_cuff_ 20–30 cmH_2_O			
Yes^a^	18 (100)	18 (100)	NA
% of time^b^	34 (17, 57)	50 (35, 64)	0.184
*P*_cuff_ <20, cmH_2_O			
Yes^a^	17 (94)	18 (100)	>0.999
% of time^b^	23 (5, 63)	43 (16, 60)	0.528
*P*_cuff_ >30, cmH_2_O			
Yes^a^	18 (100)	16 (89)	0.500
% of time^b^	26 (14, 40)	7 (1, 18)	0.002

Data are frequencies (%) or median (interquartile range)

*P*_*cuff*_ cuff pressure, *NA* not applicable

^a^Yes indicates the number of patients with at least one *P*_cuff_ 20–30, >20, or >30 cmH_2_O

^b^% of time indicates all the time spent with *P*_cuff_ 20–30, >20, or >30 cmH_2_O reported to the total recording time during each study period

### Impact of the mechanical device on *P*_cuff_

No significant difference was found in mean *P*_cuff_, percentage of time spent with *P*_cuff_ between 20 and 30 cmH_2_O, percentage of time spent with *P*_cuff_ <20 cmH_2_O, or coefficient of variation of *P*_cuff_. The percentage of time spent with *P*_cuff_ >30 cmH_2_O was significantly higher during continuous control compared with routine care of tracheal cuff (Table 2). No significant difference was found in the percentage of patients with *P*_cuff_ 20–30 cmH_2_O, *P*_cuff_ <20 cmH_2_O, or *P*_cuff_ >30 cmH_2_O during continuous control of *P*_cuff_ and routine care (Table 2). No significant difference was found in the number of underinflation episodes between continuous control and routine care periods (median (IQR) 0 (0, 5) versus 2 (0, 7), *p* = 0.733).

### Subgroup analysis

After exclusion of the four patients with high mean airway pressure (>17 cmH_2_O), no significant difference was found in mean *P*_cuff_ (26 (18, 28) versus 22 (19, 27) cmH_2_O, *p* = 0.177), percentage of time spent with *P*_cuff_ between 20 and 30 cmH_2_O (50 (24, 58) versus 53 (24, 71), *p* = 0.124), percentage of time spent with *P*_cuff_ <20 cmH_2_O (16 (2, 51) versus 35 (9, 51), *p* = 0.3), or coefficient of variation of *P*_cuff_ (15 (10, 22) versus 16 (10, 24), *p* = 0.363). The percentage of time spent with *P*_cuff_ >30 cmH_2_O was still significantly higher during continuous control compared with routine care of tracheal cuff (31 (18, 42) versus 8 (3, 20), *p* = 0.006).

## Discussion

The results of our study suggest that PressureEasy® was not more efficient in maintaining *P*_cuff_ within the recommended range (20–30 cmH_2_O) than routine care of tracheal cuff using a manometer every 4 h. No significant difference was found between continuous control of *P*_cuff_ using the mechanical device and routine care regarding *P*_cuff_, percentage of time spent with underinflation of tracheal cuff, and coefficient of variation of *P*_cuff_. However, the percentage of time spent with overinflation of tracheal cuff was significantly higher during continuous control compared with routine care.

One potential explanation for this result is the high frequency of *P*_cuff_ control using the manometer, i.e., every 4 h, during routine care. A recent prospective study performed in a cohort of 102 patients receiving invasive mechanical ventilation and manual control of *P*_cuff_ using a manometer every 8 h found a lower percentage of time (18 %) spent within the target range [14]. However, this explanation is unlikely because our results are in agreement with those of another recent trial, in which *P*_cuff_ was controlled using a manometer every 8 h [13]. Further, previous studies showed that each time a manometer is connected to the tracheal cuff, a sudden drop of *P*_cuff_ is observed [22, 26]. This is probably related to transient underinflation of tracheal cuff and might promote microaspiration of contaminated secretions [27]. Therefore, controlling *P*_cuff_ more frequently in order to maintain it within the target range could probably not be recommended.

In spite of strictly applying instructions for user of the mechanical device, the percentage of time spent within the target range of *P*_cuff_ was only 34 %. Previous studies using different devices to continuously control *P*_cuff_ reported better performances and a percentage within the targeted range >90 % [19, 20, 22, 28]. Another potential explanation for the low efficiency of the device is the mechanism of *P*_cuff_ control. This device uses the respiratory flow to inflate tracheal cuff during inspiration. Therefore, it is dependent on respiratory flow and airway pressure that may widely vary in critically ill patients. In addition, the key principle is probably an equilibrium between airway pressure and *P*_cuff_, which means that the time constant of the system might play a role in the higher *P*_cuff_ [29, 30]. Further, the exact *P*_cuff_ target, using the mechanical device, could not be precisely determined or modified. In order to test the hypothesis that our results could be explained by the bad performance of the device in patients with high airway pressures, we repeated all statistical analyses after exclusion of patients with the highest mean airway pressure but found similar results. This might be explained by the fact that high airway pressure could have occurred during only a short period of the total recording time and the relatively small number of included patients.

To our knowledge, our study is the first to evaluate the PressureEasy® mechanical device, and its results suggest that the device should not be used in critically ill patients. Our study also raises the important question of why medical devices such as tracheal tubes or *P*_cuff_ controllers could obtain the *Communauté Européenne* (CE) mark and be used in critically ill patients without any published clinical data proving their efficiency. Nevertheless, clinical evaluation before CE mark could be very complex and might reduce the development of new technologies.

No significant difference was found in coefficient of variation of *P*_cuff_ between the two study periods. However, *P*_cuff_ variation in study patients was relatively small and had probably no clinical impact, except in those patients with *P*_cuff_ around 20 or 30 cmH_2_O.

Some limitations of our study should be acknowledged. First, this was a single-center study. All study patients received assist-control ventilation, and a high proportion of them were sedated. Therefore, our results may not be generalized to patients in other ICUs, especially to nonsedated patients receiving pressure support ventilation. Second, the number of studied patients was relatively small. However, *P*_cuff_ was continuously recorded for 48 h. In addition, the number of included patients was calculated based on our hypothesis before starting the study. Third, because of the study design, namely the fact that every patient was its own control, we could not evaluate complications related to underinflation or overinflation of tracheal cuff. However, we think that this design is probably optimal as a first step in order to validate the device, before performing larger studies evaluating its impact on complications. Our study design allowed adjustment of patient-related confounding factors, such as tracheal anatomy, tracheal tube size, and airway pressure. Fourth, we excluded patients who could not be included during the first 48 h of their invasive mechanical ventilation. This exclusion criterion was selected because duration of intubation was identified as a risk factor for underinflation of tracheal cuff [14]. Fifth, we did not collect *P*_cuff_ values obtained manually during the routine care period and did not evaluate the relationship between manometer connection and any drop in *P*_cuff_. However, several previous well-designed and performed studies have clearly confirmed this relationship [22, 26]. Finally, Glascow Coma score was used to evaluate consciousness in study patients. However, a sedation score would have been more appropriate in sedated patients.

## Conclusions

The PressureEasy® device did not demonstrate a better control of *P*_cuff_ within the target range (20–30 cmH_2_O), compared with routine care using a manometer every 4 h. Moreover, the percentage of time spent with overinflation of tracheal cuff was more frequent using this device compared with routine care, which might increase the risk for tracheal ischemic lesions. Therefore, the use of this device could not be recommended in critically ill patients. Further large studies are required to confirm our results.

## Abbreviations

CE: Communauté Européene
HPLV: high-volume low-pressure
*P*_cuff_: cuff pressure
PEP: positive expiratory pressure
SAPS: simplified acute physiology score
SOFA: sequential organ failure assessment
PVC: polyvinyl chloride
